# Concurrent Validity of the Static and Dynamic Measures of Inspiratory Muscle Strength: Comparison between Maximal Inspiratory Pressure and S-Index

**DOI:** 10.21470/1678-9741-2019-0269

**Published:** 2020

**Authors:** Guilherme de Souza Areias, Luan Rodrigues Santiago, Daniel Sobral Teixeira, Michel Silva Reis

**Affiliations:** 1 Programa de Pós-Graduação em Educação Física e Desportos, Universidade Federal do Rio de Janeiro – UFRJ, Rio de Janeiro, RJ, Brazil.; 2 Grupo de Pesquisa em Avaliação e Reabilitação Cardiorrespiratória – GECARE, Departamento de Fisioterapia, Universidade Federal do Rio de Janeiro – UFRJ, Rio de Janeiro, RJ, Brazil.; 3 Programa de Pós-Graduação em Ciências Cardiovasculares, Universidade Federal do Rio de Janeiro – UFRJ, Rio de Janeiro, RJ, Brazil.

**Keywords:** Residual Volume, Maximal Respiratory Pressures, Linear Models, Total Lung Capacity, Muscle Strength, Muscle Contraction

## Abstract

**Objective:**

To verify the concurrent validity between the inspiratory muscle strength (IMS) values obtained in static (maximal inspiratory pressure [MIP]) and dynamic (S-Index) assessments.

**Methods:**

Healthy individuals were submitted to two periods of evaluation: i) MIP, static maneuver to obtain IMS, determined by the Mueller’s maneuver from residual volume (RV) until total lung capacity (TLC); ii) and S-Index, inspiration against open airway starting from RV until TLC. Both measures were performed by the same evaluator and the subjects received the same instructions. Isolated maneuvers with differences < 10% were considered as reproducible measures.

**Results:**

Data from 45 subjects (21 males) were analyzed and that showed statistical difference between MIP and S-Index values (133.5 ± 33.3 and 125.6 ± 32.2 in cmH2O, respectively), with *P*=0.014. Linear regression showed *r^2^*=0.54 and S-Index prediction formula = 39.8+(0.75×MIP). Pearson’s correlation demonstrated a strong and significant association between the measures with *r*=0.74. The measurements showed good concordance evidenced by the Bland-Altman test.

**Conclusion:**

S-Index and MIP do not present similar values since they are evaluations of different events of the muscular contraction. However, they have a strong correlation and good agreement, which indicate that both are able to evaluate the IMS of healthy individuals.

**Table:** 

Abbreviations, acronyms & symbols	
BMI	= Body mass index
IMS	= Inspiratory muscle strength
IMT	= Inspiratory muscle training
IPAQ	= International Physical Activity Questionnaire
MIP	= Maximal inspiratory pressure
RV	= Residual volume
TLC	= Total lung capacity

## INTRODUCTION

S-Index is a non-invasive, resistance-free, and easy-to-apply measure for dynamically assessing the inspiratory muscle strength (IMS)^[1]^. In theory, the S-Index is able to support the inspiratory muscle training (IMT) and is becoming popular due to its easy applicability and low cost, since the same device is able to provide IMS and inspiratory muscle resistance evaluation, in addition to the IMT. Besides that, comparing to the maximal inspiratory pressure (MIP), a better adaptation of individuals to the evaluation was reported, since a more functional maneuver is performed, mimicking the physiological contraction of the inspiratory muscles^[2,3]^.

MIP is a parameter obtained through manovacuometry and is commonly used in clinical practice to assess general respiratory muscle function in a static manner. Manovacuometry is widely described in the literature as a maneuver that requires the evaluated individual to perform a maximal contraction, starting from the residual volume (RV) until a total lung capacity, in order to generate great isometric effort of the inspiratory musculature. Alternatively, S-Index evaluates the specific muscle function, in a dynamic way, through the flow generated in the open system^[4,5]^.

In this sense, given the particularities of biomechanics and muscular architecture innervation of the diaphragmatic muscles, the following questions can be raised: Do IMS static measures adequately characterize the ability of the inspiratory muscles to generate force even though they do not mimic the physiological diaphragmatic incursion? Are static assessments similar to dynamic inspiratory muscle assessments?

The hypothesis of this study is that the dynamic measurement of the IMS, obtained through S-Index, is valid as an alternative tool to MIP. In this scenario, the objective of this study was to verify the concurrent validity between the IMS values obtained through static (MIP) and dynamic (S-Index) assessments.

## METHODS

### Study Design

This is a prospective, cross-sectional, and randomized study.

### Subjects

Healthy individuals, of both genders, and aged between 18 and 40 years were recruited. Individuals with a history of smoking, drug use, cardiovascular diseases (such as systemic arterial hypertension, heart failure, electrical conduction disorder, among others), respiratory disease (obstructive or restrictive impairment), and muscular (myopathy), neurological, metabolic (diabetes mellitus), or immune disorders were excluded. Individuals who did not adapt to any of the devices during the period of familiarization or who presented inspiratory muscle weakness according to the prediction formulas proposed by Neder et al.^[4]^ (1999) of MIP were also excluded. The Research Ethics Committee of the Hospital Universitário Clementino Fraga Filho of the Universidade Federal do Rio de Janeiro (CAAE 43656115.8.0000.5257/2015) approved this study. The volunteers signed an informed consent form to participate in the research.

### Screening

At first, the subjects were submitted to anamnesis and physical examination, in order to investigate their history of previous diseases as well as their lifestyle. A detailed evaluation was applied, in which the personal data, anthropometrics, and vital signs were collected. The short form of the International Physical Activity Questionnaire (IPAQ) was used in order to stratify the level of physical activity into very active, active, irregularly active, and inactive^[6]^.

### Inspiratory Muscle Strength Assessment

IMS evaluation was always performed by the same evaluator, with the volunteer at rest and in the sitting position. The order of the devices was randomized to all subjects using an opaque envelope. In both tests, volunteers were instructed not to perform compensatory head and trunk movements that could cause bias in the assessment.

### Familiarization

Initially, the volunteers were submitted to a warm-up and familiarization stage with the devices and maneuvers to be performed^[7]^. Between each of the maneuvers performed, a period of 30 seconds was given respecting the rest of the diaphragmatic muscles to avoid bias in subsequent maneuvers^[8,9]^. For proper performance of each test and consequent learning, the volunteer should be in a sitting position with his back resting on the chair, avoiding head and trunk movements. The instructions for each device were followed in the IMS test in order to simulate the effort and the actual maneuver to be performed. To qualify the period of familiarization, the following criterion was considered: evaluation of the trained physiotherapist, observing if the individual was able to follow the previously established guidelines and if the volunteer was able to adapt to the linear inspiratory electronic resistor and the isometric inspiratory vacuum deformation resistor. A maximum period of 15 minutes for each method was tolerated for this learning. In case of a non-successful familiarization, the volunteer was excluded from the research.

### S-Index

A nasal clip and a linear inspiratory resistor (PowerBreatheK5, IMT Technologies Ltd., Birmingham, UK) were adapted to allow measurement of the S-Index. The measurement was determined after maximal inspiratory effort from the RV against the buccal properly connected to the voluntary, having a strong verbal stimulus provided by the evaluator. Intervals of 30 seconds between each maximal effort were respected in order to avoid fatigue of the musculature involved^[9]^. The S-Index was considered the highest point of the pressure x time graph (peak pressure), obtained with Breathe Link 1.1 software, with reproducible value (difference < 10%) between three isolated efforts.

### Maximal Inspiratory Pressure

A nasal clip and an isometric inspiratory with manovacuometer device (MVD 300, GlobalMed, Porto Alegre, Brazil) were adapted to allow the measurement of MIP. This was determined after maximal inspiratory effort from RV against the buccal properly connected to the volunteer, having a strong verbal stimulus granted by the evaluator. MIP was the highest reproducible value (maintained for at least one second – plateau pressure – and with a difference of < 10% between three isolated efforts). Intervals of 30 seconds between each maximal effort were respected to avoid fatigue of the involved musculature^[9]^. The values of prediction and normality were based on the regression equation proposed by Neder et al.^[4]^ (1999) for the Brazilian population and values of static pressure < 70% of the predicted were considered inspiratory muscle weakness.

### Statistical Analysis

Initially, the sample calculation was performed based on the article by Minahan et al.^[1]^. Thus, for a power of 80%, with size 5 effect, and 5% alpha, it was determined a need of 34 individuals (GPower 3.0.1.0 for Windows). Additionally, the Shapiro-Wilk test was performed to verify the data distribution and the Levene’s test was performed to assess the homogeneity of variances. Then, the paired *t*-test was applied to compare the values of S-Index and MIP. The Pearson’s correlation and the Bland-Altman tests were performed with a 95% confidence interval to evaluate the agreement between the methods. In addition, a linear regression was applied aiming to establish a prediction formula for the S-Index from the MIP for this population. The analyses were performed with Sigmaplot Software 12.0, with an established significance level of *P*<0.05.

## RESULTS

Forty-nine young and eutrophic individuals were recruited, and four of them were excluded during the familiarization process. Most subjects presented an active lifestyle according to IPAQ. None of the individuals had inspiratory muscle weakness, as shown in Table 1.

**Table 1:** Demographics and anthropometric level of physical activity and inspiratory muscle strength data.

Characteristic (n= 45)	Males (n=21)	Females (n=24)
Age (y)	21.86±2.59	22.08±2.95
Weight (kg)	73.71±11.55	57.25±8.98
Height (m)	1.76±0.08	1.63±0.06
BMI (kg/m^2^)	23.67±3.51	21.45±3.02
IPAQ, very active (%)	52.3	29.2
IPAQ, active (%)	38.1	41.6
IPAQ, irregulary active (%)	3.6	20.8
IPAQ, inactive (%)	0	8.4
Predicted MIP (cmH_2_O)	137.81±2.08	99.58±1.44
MIP (cmH_2_O)	146.33±22.52	111.42±25.31
S-Index (cmH_2_O)	159±20.11	115.29±21.27

Data expressed as mean ± standard deviation or percentages. (kg)=kilograms; (kg/m^2^)=kilograms per square meters; (m)=meters; (y)=years. BMI=body mass index; IPAQ=International Physical Activity Questionnaire; MIP=Maximal inspiratory pressure

**Fig. 1:**
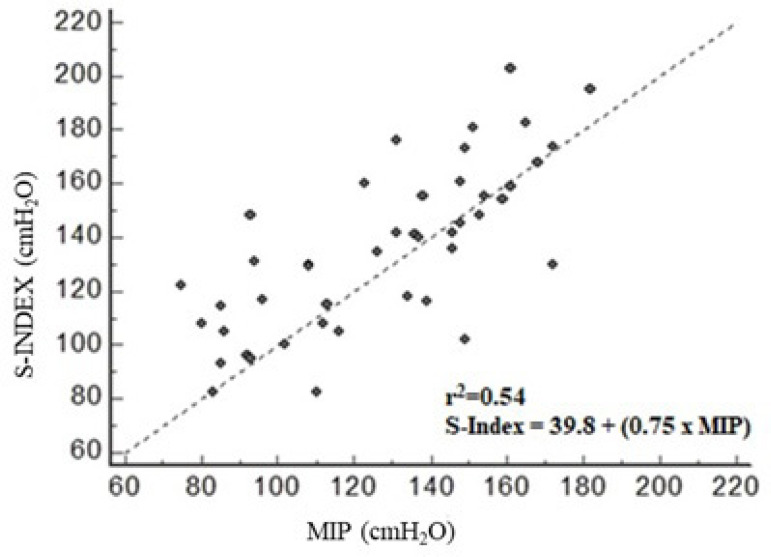
Pearson's correlation and determination coefficient between maximal inspiratory pressure (MIP) and S-Index: r=0.74 with P<0.001.

In the comparison between S-Index and MIP, a significant difference was observed (133.5 ± 33.3 and 125.6 ± 32.2 in cmH_2_O, respectively), with *P*=0.014. Pearson’s correlation showed a strong association between MIP and S-Index measurements with *r*=0.74 and *P*<0.0001. In addition, the linear regression analysis showed an *r*^2^=0.54 and the prediction equation: S-Index = 39.8 + (0.75 x MIP) (Figure 1).

**Fig. 2:**
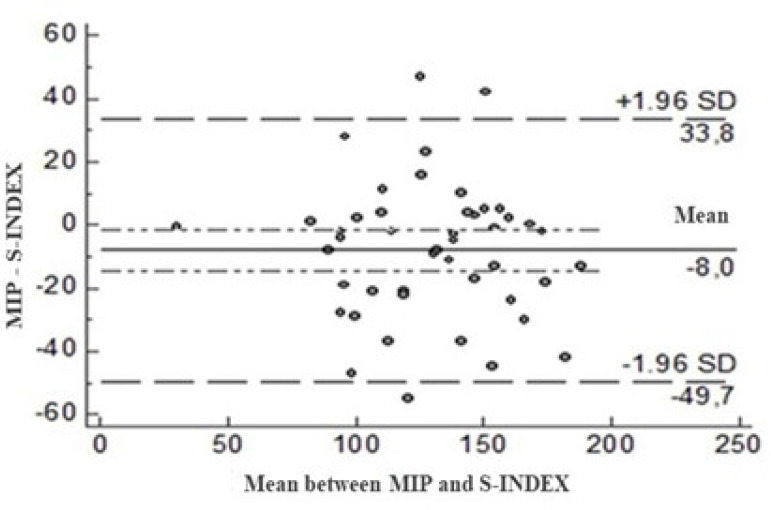
Bland-Altman concordance for maximal inspiratory pressure (MIP) and S-Index values.

Bland-Altman’s agreement analysis revealed that MIP and S-Index values showed a symmetric distribution around the midline, confirming the good agreement of S-Index with MIP (Figure 2).

## DISCUSSION

The main findings of this study indicated that the values obtained from S-Index and MIP are significantly different. However, a strong Pearson’s correlation and a Bland-Altman’s analysis revealed good agreement between the methods. This study was pioneer in comparing the measures of MIP with S-Index using a robust method, and that problematizes questions that associate the specificity of inspiratory muscle function to the type of contraction performed (static and dynamic) in each evaluation method.

Therefore, a homogeneous sample of apparently healthy subjects was selected. This may have minimized confounding factors that could interfere with measured variables, such as muscle or lung function. Then, a familiarization period was allowed considering the learning effect for both static and dynamic evaluations. Finally, in order to guarantee the reproducibility of the measurements, the instructions and maneuvers were guided by the same verbal command and evaluator and executed in the same position (sitting).

Inadvertently, the S-Index has been confused with MIP to evaluate IMS. In this sense, Lee et al.^[2]^ (2016) revealed a high correlation coefficient of IMS intra- and inter-rater (0.986 and 0.984, respectively) in their study. They used the PowerBreathe K5 device to obtain those results; however, this device evaluates muscle strength at flow with an evaluation of the dynamic muscle contraction. Thus, the authors could not infer that the values obtained were MIP, as described in their study. The same error was observed in the study by Salazar-Martinez et al.^[3]^ (2017), who also evaluated IMS dynamically through the PowerBreathe K3 device, where they aimed to observe the influence of IMT on ventilatory efficiency and impact during cycling activity in normoxia and hypoxia. The authors also assumed that the dynamic measure of force was MIP.

Although MIP is a tool established in the IMS benchmarking literature, the technical characteristics of its implementation do not reflect the measure obtained by S-Index. This is because MIP is obtained through the manovacuometer, a device that calculates IMS based on a plateau of negative pressure obtained through the Mueller’s maneuver and affects the individual's ability to perform a static forced inspiration against an occluded pathway^[4,10,11]^. The S-Index analysis takes place through the flow, where the individual performs a dynamic, rapid deep inspiration against a linear load resistor that converts the flow data into pressure. Because the method of obtaining blood pressure values is appropriate in each device and the lack of previous validation studies, it would not be possible to infer the similarity between the measures that allowed S-Index to be analyzed and called MIP. Nor are studies validating S-Index as a measure capable of reliably analyze IMS, which was demonstrated in our study in a pioneering way.

Minahan et al.^[1]^ (2015) demonstrated similar results to our study when they proposed S-Index as a valid IMS assessment. However, the results of these authors are not compatible with our study once they found a poor correlation between the devices (r=-0.35). In this aspect, there are methodological considerations such as i) the familiarization with the devices and the maneuver is unclear; ii) low number of individuals. This corroborates the importance of more robust methods for comparing measures of MIP and S-Index, as applied by our study.

An important discussion about muscle physiology and biomechanics concerns the generality and specificity of strength assessments. The generalist line believes in a general force component where any assessment of force, whether it is isometric, isotonic, or isokinetic, would be able to reflect the strength of more and less strong individuals according to Hortobagyi, Lachance, and Katch, 1987, *apud*, Baker, Wilson, and Carlyon, 1994^[12]^. However, if there is a general force component, different methods of evaluation, velocities, and modes of muscle contraction should not be considered, since any method of force evaluation would be able to stratify it. However, another line looks for specific strength assessments where dynamic strength evaluation would be able to elucidate only dynamic and functional muscle capacities and isometric strength assessments would be predictors of just isometric muscle capacities^[12]^.

There are studies for different muscle groups demonstrating that static evaluations are not able to predict functional muscular capacities. Feeler, James, and Schapmire (2010)^[13]^ evaluated the elevation of legs, arms, and back of non-sedentary workers and the static evaluation test was not able to accurately reflect the dynamic lifting functions. Murphy and Wilson (1996)^[14]^ performed static elbow flexion tests at different angles (90° and 120°) with electromyography data from triceps and pectoralis major of healthy men and compared with the specific function of throwing a medicinal ball. The authors observed poor correlations at both flexion angles when compared to functional activity. The electromyographic data also indicated that there were differences in the neural recruitment of fibers between the activities, which raises the hypothesis of activation of different muscle motor units, making it impossible to static tests to evaluate the function of the muscles of the upper limbs. Thomas et al.^[15]^ (2015) evaluated the vertical jump of young male athletes. Using a force platform, they obtained power and peak force variables, isometric force peak, and isometric mean peak and those were compared with the height obtained in the test. There was no correlation between isometric scores and jump height since stronger athletes did not jump higher than the weaker athlete did.

Another point to be emphasized is the hemodynamic repercussion that the Mueller’s maneuver can generate on the cardiovascular system. For some special groups, this may have a higher associated risk. Scharf et al.^[16]^ (1987) demonstrated that there is left ventricular akinesia in individuals with coronary artery disease or previously infarcted during multiple forced inspirations against an occluded airway. Sampol et al.^[17]^ (2003) observed that high intrathoracic negative pressures generate increased sympathetic activity by increasing the incidence of aortic dissection in individuals with obstructive sleep apnea and Marfan syndrome. Also, some studies showed the need to perform a previous familiarization because MIP maneuver is volitional and not very intuitive^[7,18,20,21]^. Thus, in addition to the evaluation rate plus the familiarization, it can be inferred an increase in cardiovascular risk for the use of the MIP maneuver in these populations, which would be minimized through IMS evaluation in a dynamic way.

In this context, a hypothesis was raised that for optimal evaluation of muscle function, dynamic assessments of strength should be prioritized, which raises the question: Is the static contraction obtained through MIP able to support the workload of IMT? Reflecting on the question, it is objected that a static contraction may not adequately categorize dynamic training since currently the most usual way to perform IMT is through dynamic load-contractions with linear load^[19,22]^. Based on the results of this study, there is a difference between the values of static and dynamic contractions, and this differentiation may be related to the generality presented by MIP when evaluating IMS that contrasts with the S-Index specificity for the same task^[12]^. The strong correlation and concordance between these evaluations suggest that both measures are used to evaluate IMS in a complementary way. However, there is not yet enough data to corroborate the S-Index as a predictor of morbidity of any pathology, nor that it is an index able to predict muscle weakness, as occurs with MIP. Finally, it is noteworthy that, although the evaluation of muscle strength through manovacuometry is widespread, S-Index has been obtained in the same device capable of being applied for IMS. This has to do with good practices associated with therapeutic management since it makes cost-effectiveness feasible.

### Methodological Considerations

Due to the different methods of collection for each evaluation, some individuals were unable to reach their MIP values in one of the evaluations. This occurred because of the inability of some individuals to inhale against resistance (MIP) or in absence of it (S-Index). Those individuals were subsequently excluded during the period of familiarization.

## CONCLUSION

The results of this study promote the S-Index as a tool capable of evaluating IMS of healthy individuals. Therefore, when assessed by S-Index, this measure could be more specific for evaluating respiratory muscle strength and, consequently, more appropriate for the prescription of IMT.

**Table:** 

Author's roles & responsibilities	
GSA	Substantial contributions to the conception or design of the work; or the acquisition, analysis, or interpretation of data for the work; drafting the work or revising it critically for important intellectual content; final approval of the version to be published
LRS	Substantial contributions to the conception or design of the work; or the acquisition, analysis, or interpretation of data for the work; final approval of the version to be published
DST	Drafting the work or revising it critically for important intellectual content; final approval of the version to be published
MSR	Substantial contributions to the conception or design of the work; or the acquisition, analysis, or interpretation of data for the work; drafting the work or revising it critically for important intellectual content; agreement to be accountable for all aspects of the work in ensuring that questions related to the accuracy or integrity of any part of the work are appropriately investigated and resolved; final approval of the version to be published
